# N‐3 Polyunsaturated Fatty Acids to Prevent Atrial Fibrillation: Updated Systematic Review and Meta‐Analysis of Randomized Controlled Trials

**DOI:** 10.1161/JAHA.112.005033

**Published:** 2013-02-22

**Authors:** Javier Mariani, Hernán C. Doval, Daniel Nul, Sergio Varini, Hugo Grancelli, Daniel Ferrante, Gianni Tognoni, Alejandro Macchia

**Affiliations:** 1 Fundación GESICA (Grupo de Estudio de Investigación Clínica en Argentina), Buenos Aires, Argentina (J.M., H.D., D.N., S.V., H.G., D.F., A.M.); 2 Fondazione Mario Negri Sud, Santa Maria Imbaro (CH), Italy (G.T., A.M.)

**Keywords:** arrhythmia, atrial fibrillation, fatty acids, meta‐analysis, prevention

## Abstract

**Background:**

Previous studies have suggested that n‐3 polyunsaturated fatty acids (n‐3 PUFAs) have antiarrhythmic effects on atrial fibrillation (AF). We aimed to assess the effects of therapy with n‐3 PUFAs on the incidence of recurrent AF and on postoperative AF.

**Methods and Results:**

Electronic searches were conducted in Web of Science, Medline, Biological Abstracts, Journal Citation Reports, and the Cochrane Central Register of Controlled Trials databases. In addition, data from the recently completed FORωARD and OPERA trials were included. We included randomized controlled trials comparing treatment with n‐3 PUFAs versus control to (1) prevent recurrent AF in patients who underwent reversion of AF or (2) prevent incident postoperative AF after cardiac surgery. Of identified studies, 12.9% (16 of 124) were included, providing data on 4677 patients. Eight studies (1990 patients) evaluated n‐3 PUFA effects on AF recurrence among patients with reverted AF and 8 trials (2687 patients) on postoperative AF. Pooled risk ratios through random‐effects models showed no significant effects on AF recurrence (RR, 0.95; 95% CI, 0.79 to 1.13; I^2^, 72%) or on postoperative AF (0.86; 95% CI, 0.71 to 1.04; I^2^, 53.1%). A funnel plot suggested publication bias among postoperative trials but not among persistent AF trials. Meta‐regression analysis did not find any relationship between doses and effects (*P*=0.887 and 0.833 for recurrent and postoperative AF, respectively).

**Conclusions:**

Published clinical trials do not support n‐3 PUFAs as agents aimed at preventing either postoperative or recurrent AF.

**Clinical Trial Registration:**

URL: http://www.crd.york.ac.uk/PROSPERO. Unique Identifier: CRD42012002199.

## Introduction

Atrial fibrillation (AF) is the most common arrhythmia in adults, and its incidence is increasing worldwide.^1–2^ Classic antiarrhythmic drugs used to preserve normal sinus rhythm in patients with previous AF have shown limited efficacy as well as frequent and serious harmful effects.^3–5^ For this reason, actively searching for antiarrhythmic agents without the common adverse events of classic antiarrhythmic drugs has become increasingly important.^6–9^ Although statins and angiotensin II receptor blockers may favorably affect the atrial remodeling associated with AF,^6–8^ the results of clinical trials have been neutral.^9–11^ In addition, new antiarrhythmic drugs proved to be neither more effective nor safer than classic therapies.^12^

N‐3 polyunsaturated fatty acids (PUFAs) from animal sources have shown antiarrhythmic properties on ventricular arrhythmia in patients with previous myocardial infarction,^13–14^ although recent findings failed to replicate these results.^15–16^ Encouraged by previous basic,^17–18^ epidemiological,^19–20^ and clinical data ^13–14^ on ventricular arrhythmias, a body of experimental data has suggested a potential role of these compounds in treating atrial arrhythmias.^21–22^ Clinical trials have focused their attention on 2 different populations: patients with persistent/paroxysmal AF for whom the principal objective is to preserve normal sinus rhythm after reversion and patients who have undergone cardiac surgery to prevent the onset of new AF. Overall, the results of clinical trials led to conflicting results. Previous systematic reviews failed to provide a definitive answer because the numbers of patients and events were relatively small,^23–25^ but since their publication, the number of patients available for assessment has more than doubled. Therefore, we have conducted a systematic review and meta‐analysis to evaluate the effects of n‐3 PUFAs on sinus rhythm maintenance after AF reversion and on AF incidence after cardiac surgery.

## Methods

The protocol for our study is registered in the international prospective register of systematic reviews (PROSPERO). Registration number CRD42012002199 (available from http://www.crd.york.ac.uk/PROSPERO/display_record.asp?ID=CRD42012002199). This systemic review is reported following recommendations of the PRISMA statement.^26^

### Eligibility Criteria

To be included in the meta‐analysis, studies had to be randomized controlled trials evaluating any dose and formulation of n‐3 PUFAs, administered as pharmacological preparations and conducted in either of the following settings: sinus rhythm maintenance after spontaneous electrical or pharmacological cardioversion or AF prevention in patients undergoing cardiac surgery.

Studies could be double‐blind, placebo‐controlled, or unexposed controlled trials. In sinus rhythm maintenance trials, patients could be randomized with AF or in sinus rhythm (ie, before or after reversion).

In cardiac surgery trials, all patients had to be in sinus rhythm at randomization. No restriction criterion on type of surgery was adopted.

We excluded nonrandomized studies, those that did not reported data on atrial fibrillation occurrence during follow up, those with no follow‐up (ie, evaluating the electrophysiological effects of 1 or few doses of n‐3 PUFAs), and those that were reported in languages other than English.

Trials reported as proceeding abstracts were included if other inclusion criteria were met.

### Search Strategy

We conducted an electronic search in the Web of Science database, simultaneously searching in the Web of Science database (from 1972 to November 6, 2012), Medline (from 1950 to November 6, 2012), Biological Abstracts (from 1995 to November 6, 2012) and Journal Citation Reports. We also electronically searched the Cochrane Central Register of Controlled Trials database.

Search terms were “(n‐3 PUFA OR n 3 polyunsaturated OR fatty acids OR fish oil OR docosahexaenoic OR eicosapentaenoic OR polyunsaturated fatty acids) AND (atrial fibrillation OR atrial flutter) AND (random* OR randomised OR randomized),” searched in titles or as topics.

Additional searches included reference lists of relevant articles and reference lists of previous systematic reviews on this topic. Data from the recently released FORωARD (Fish Oil Research with ω‐3 for Atrial fibrillation Recurrence Delaying, Trial Registration Identifier: NCT00402363) trial were also included in the analyses.

### Data Collection

Two investigators (J.M. and A.M.) independently collected information from studies retrieved by the initial search in an unblinded fashion. Titles and abstracts were scrutinized to check eligibility, and when inclusion and exclusion criteria were unclear, the full text report was evaluated.

Data abstracted from the included studies were authors, date of publication, design, comparator, dosage and formulation of n‐3 PUFA, loading dose, recurrent/incident AF definition, number of participants, patient characteristics, target population (ie, persistent AF or postoperative AF), type of surgery, and outcomes of interest. Abstracted data were collected in paper form and then entered in a database designed for the study. All discrepancies were solved by consensus with a third investigator (D.F.). No attempt was made to standardize definitions of end points. Quality of the studies was assessed using the score suggested by Jadad et al.^27^

### Outcomes

The primary outcome was the occurrence of AF. For persistent AF studies, this was recurrent AF, and for postoperative studies, incident AF. Secondary outcomes were all‐cause mortality and length of ICU stay (only for postoperative studies).

### Statistics

All analyses were conducted separately for trials of persistent AF and postoperative AF. For every study, we computed risk ratios and corresponding 95% confidence intervals (CIs) for outcomes in the n‐3 PUFA group compared with control/placebo group. Risk ratios from each individual trial were pooled using the random‐effects model approach as described by DerSimonian and Laird.^28^ For postoperative AF trials, we also computed mean length of ICU stay and pooled all means using weighted mean differences (also with a random‐effects model).

Heterogeneity was assessed through the Cochran Q test, with a *P*<0.1 indicating statistically significant heterogeneity. To further measure heterogeneity, inconsistency (ie, the I^2^ statistic) was computed, considering >50% as moderate inconsistency.^29^ Additional prespecified analyses to explore possible sources of heterogeneity between studies include a repeated pooled analysis excluding studies with less than the median quality score. Other sensitivity analyses by β‐blocker therapy, amiodarone therapy, age (≤ or >median), and sex were conducted. To further explore potential sources of heterogeneity in the estimated effect sizes between studies, we conducted meta‐regression analyses, in which the dependent variable (the [log] risk ratios) was weighted‐regressed against covariates at the study level (n‐3 PUFA dose, AF rate in the control group, quality score, mean age, proportion who were male, rate of β‐blocker and amiodarone use at baseline, left ventricular ejection fraction). Meta‐regression was conducted separately for recurrent AF studies and postoperative AF studies.^30^ Publication bias was evaluated using visual inspection of the funnel plot and Egger test, with *P*<0.1 indicating evidence of statistically significant asymmetry in the funnel plot.^31^

## Results

### Included Studies

**Figure 1.**
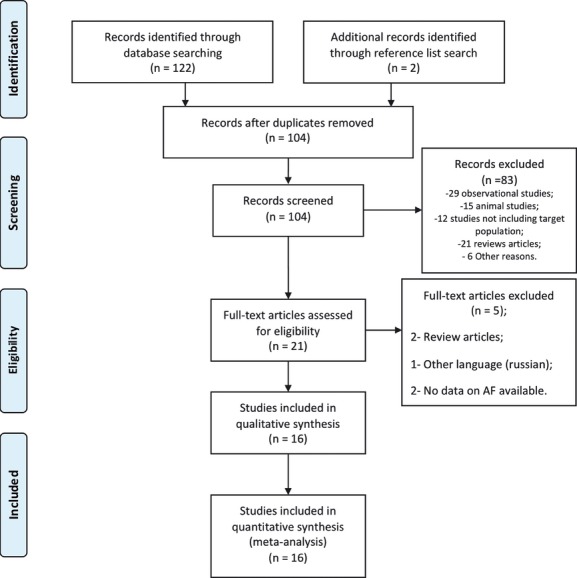
Studies flow.

The electronic search identified 122 potentially eligible reports, of which 20 were eliminated as duplicates. After title and abstract assessment of the remaining 102 reports, 83 reports were excluded. Two additional studies were identified in the reference lists of relevant articles, yielding 21 reports that were retrieved for full‐text evaluation. Five additional studies were excluded, leaving 16 for our analysis^32–47^: 13 studies as journal articles and 3 as proceeding abstracts^32–33^ (Figure 1).

Table 1 shows the characteristics of the studies. Overall, 4712 patients were included in the studies, but 35 patients were excluded from 2 trials,^34–35^ leaving 4677 patients for analyses. Eight studies (n=1990 patients) evaluated n‐3 PUFA effects on AF recurrence among patients with reverted persistent or paroxysmal AF.^32–39^ The other 8 trials (n=2687 patients) evaluated the effects on postoperative AF.^40–47^

**Table 1.** Studies' Design and Quality Score

Study	Year	Design	Inclusion Criteria	Exclusion Criteria	Follow‐up	End‐Point Definition	N‐3 PUFA Dose	Ratio EPA/DHA	Assessment of Outcomes	Jadad's Score
Persistent or paroxysmal atrial fibrillation										
Erdogan et al^32^	2007	Triple blind	Persistent AF scheduled for external cardioversion	Cardiac or extracardiac abnormalities causing AF (mitral stenosis, hyperthyroidism)	12 months	N/A	N/A	N/A	N/A	—
Margos et al^33^	2007	Open label	Cardioverted persistent AF, euthyroid, and under anticoagulation	LVEF ≤40%, LA >55 mm or at least moderate valvular heart disease	6 months	Persistent AF	N/A	N/A	24‐hour Holter at 1 month and ECG at 1, 3, and 6 months	—
Kowey et al^36^	2010	Double blind	Sinus rhythm and ≥1 suspected or documented episode of AF in the last 3 months and ≥1 documented episode of AF in the last 12 months.	Permanent AF, secondary AF, structural cardiac disease, use of antiarrhythmic drugs (class I or III, amiodarone in the last 6 months)	6 months	Symptomatic recurrence of AF or flutter among paroxysmal AF patients. Symptomatic recurrence of AF or flutter among all patients was a secondary outcome.	3.4 g/day	1.24/1	Transtelephonic monitoring and ECG	5
Bianconi et al^35^	2011	Double‐blind	Persistent AF lasting more than 1 month and scheduled for electrical cardioversion	Use of n‐3 PUFA, MI in the last 3 months, uncompensated heart failure	6 months	AF recurrence	1.7 g/day	1.2/1	Transtelephonic monitoring and ECG	5
Özaydin et al^37^	2011	Open‐label	Successful electrical cardioversion for persistent AF	Paroxysmal AF, left atrium >55 mm, moderate‐to‐severe heart valve disease, coronary artery disease, NYHA class III to IV heart failure	12 months	AF >10 minutes	0.6 g/d	1.5/1	ECG	1
Nodari et al^34^	2011	Double blind	Persistent AF lasting ≥1 month, ≥1 relapse after successful previous cardioversion	Left atrium >60 mm, severe heart valve disease, myocardial infarction in previous 6 months	12 months*	Sinus maintenance	1.7 g/day	1.2/1	ECG and 24‐hour Holter monitoring at 1, 3, 6, and 12 months	5
Kumar et al^38^	2011	Open label	Persistent AF, 18 to 85 years, scheduled for electrical cardioversion	Paroxysmal AF, left atrium >60 mm, severe heart valve disease, NYHA class IV heart failure.	12 months	Persistent AF recurrence	1.74 g/day	1.4/1	ECG	2
FORωARD^39^	2012	Double blind	≥2 Episodes of paroxysmal AF in the last 6 months (last episode within 3 months) or reverted persistent AF (within 3 to 28 days), and ≥65 years or moderate/high risk for stroke	Secondary AF, severe heart valve disease, NYHA classIV heart failure	12 months	AF recurrence	1 g/day	1/1	ECG	5
Postoperative atrial fibrillation										
Calò et al^40^	2005	Open label	Elective CABG	Valvular surgery, use of antiarrhythmic drugs (class I or III), history of supraventricular arrhythmias	In‐hospital	AF >5 minutes or requiring intervention	1.7 g/day	1:2	Continuous rhythm monitoring for 2 to 5 days and ECG	4
Heidt et al^41^	2009	Double blind	Elective CABG	Valvular surgery, use of antiarrhythmic drugs (class I or III), history of supraventricular arrhythmias	ICU stay	AF >15 minutes	100 mg/kg per day IV	N/A	Continuous rhythm monitoring and ECG	3
Heidarsdottir et al^42^	2010	Double blind	Elective or urgent cardiac surgery	<40 years, history of atrial arrhythmia, use of amiodarone or sotalol	In‐hospital (maximum 2 weeks)	AF >5 minutes	2.2 g/day	1.24/1	Continuous rhythm monitoring	3
Saravanan et al^43^	2010	Double blind	Elective isolated CABG on pump	History of atrial arrhythmias, use of antiarrhythmic drugs (class I or III) or n‐3 PUFA	In‐hospital	AF ≥30 seconds	1.7 g/day	1.2/2	Continuous rhythm monitoring for 5 days, ECG thereafter	4
Sandesara et al^44^	2012	Double blind	Elective CABG with or without valve surgery	Urgent or emergent surgery, chronic or persistent AF, use of antiarrhythmic drugs (class I or III)	2 weeks	Documented AF (ECG or rhythm strip) requiring treatment	1.7 g/day	1.24/1	Continuous rhythm monitoring during hospitalization, daily in‐hospital ECG and telephone interview	4
Sorice et al^45^	2011	Open‐label	Elective CABG	History of AF, use of antiarrhythmic drugs (class I or III), valvular surgery	In‐hospital	AF >5 minutes	1.7 g/day	1.2/1	Continuous rhythm monitoring for at least 4 days, daily ECG thereafter	1
Farquharson et al^46^	2011	Double blind	Elective CABG and/or valve surgery	Previous AF or flutter, use of antiarrhythmic drugs (class I or III), NYHA class III to IV heart failure	In‐hospital (maximum 6 days)	AF or flutter ≥10 minutes or requiring intervention	4.5 g/day	1.42/1	Continuous rhythm monitoring for 3 days, and daily ECG thereafter	5
OPERA^47^	2012	Double blind	Cardiac surgery next day of randomization or later	Absence of sinus rhythm, existing or planned cardiac transplant, or use of left ventricular assist device	In‐hospital*	AF ≥30 seconds (ECG or rhythm strip)	2 g/day	1.24/1	Continuous rhythm monitoring for ≥5 days, ECG thereafter	5

PUFA indicates polyunsaturated fatty acid; EPA, eicosapentaenoic acid; DHA, docosahexaenoic acid; AF, atrial fibrillation; N/A, not available; LVEF, left ventricular ejection fraction; LA, left atrial dimension; ECG, electrocardiogram; NYHA, New York Heart Association; CABG, coronary artery bypass grafting; ICU, intensive care unit.

* Only patients with successful electrical cardioversion or spontaneous reversion entered in the follow‐up.

* Follow‐up for mortality of 12 months.

Doses of n‐3 PUFA varied across studies from 0.6 to 4.5 g daily. All but 2 studies used an oral formulation (capsules): 1 study administered n‐3 PUFAs intravenously^41^ and the other^46^ as a liquid oil.

Among trials evaluating n‐3 PUFAs to prevent recurrence of AF after reversion, follow‐up ranged from 6 to 12 months. Assessment of outcomes included transtelephonic monitoring in 2 studies, 24‐hour Holter monitoring in 2 studies, and ECG in all studies that reported this information.

Studies evaluating n‐3 PUFAs to prevent AF after cardiac surgery had follow‐up limited to hospitalization: 1 study followed patients up to 14 days through telephone contact and the other up to 30 days.^44,47^ Methods to assess incident AF consisted of continuous monitoring for 2 to 5 days postsurgery and ECG thereafter in most studies, with only 1 study continuously monitoring patients for the complete hospital stay.^42^

Median sample size was 186 patients (189 among persistent AF studies and 181 among postoperative AF) (Table 2).

**Table 2.** Patient Characteristics

Study	N	Age,* Mean	Male Sex, n (%)	Hypertension, n (%)	Previous MI, n (%)	Diabetes, n (%)	β‐Blockers, n (%)	Amiodarone, n (%)	LA mm*, Mean	LVEF,* %
Persistent or paroxysmal atrial fibrillation										
Erdogan et al^32^	108	65.0	78 (72.2)	NA	NA	NA	NA	NA	NA	NA
Margos et al^33^	40	55.5	28 (70)	NA	NA	NA	NA	23 (57.5)	44.9	57.3
Kowey et al^36^	663*	60.5	373 (56)	NA	NA	NA	NA	0 (0)	NA*	NA*
Bianconi et al^35^	214*	69.2	129 (70)	134 (71.7)	18 (9.6)	34 (18.2)	84 (44.9)	52 (27.8)	44.9	57.7
Özaydin et al^37^	47	61.5	20 (42.6)	25 (53.2)	0 (0)	8 (17.0)	12 (25.5)	47 (100)	44	60.5
Nodari et al^34^	205*	69.5	133 (66.8)	87 (43.7)	68 (34.2)	69 (34.7)	123 (61.8)	199 (100)	46	49.5
Kumar et al^38^	182*	62.0	138 (77.5)	92 (51.7)	31 (17.4)	27 (15.2)	NA	59 (33.2)	45.8	58.4
FORωARD^39^	586	66.1	321 (54.8)	524 (91.4)	67 (11.7)	74 (12.9)	353 (60.2)	372 (63.5)	29.1*	60
Postoperative atrial fibrillation										
Calò et al^40^	160	65.6	136 (85)	128 (80)	84 (52.5)	52 (32.5)	92 (57.5)	0 (0)	39.7	55.8
Heidt et al^41^	102	64.4	70 (68.6)	NA	NA	NA	NA	0 (0)	40.3	52.2
Heidarsdottir et al^42^	168	67.0	133 (79.2)	106 (63.1)	26 (15.5)	26 (15.5)	126 (75)	0 (0)	NA	60
Saravanan et al^43^	103	66.0	82 (79.6)	33 (32)	26 (25.2)	15 (14.6)	88 (85.4)	0 (0)	NA*	NA*
Sandesara et al^44^	243	62.8	196 (80.7)	215 (88.5)	101 (41.6)	88 (36.2)	194 (80.0)	0 (0)	39.0	52.7
Sorice et al^45^	201	63.2	164 (81.6)	129 (64.2)	NA	85 (42.3)	121 (60.2)	0 (0)	40.6	52.5
Farquharson et al^46^	194	64.0	142 (73.2)	151 (77.8)	68 (35)	61 (31.4)	80 (41.2)	0 (0)	NA	64.5
OPERA^47^	1516	63.7	1094 (72.2)	1135 (74.9)	366 (24.1)	393 (25.9)	877 (57.9)	58 (3.8)	42.2	56.7

LA indicates left atrial dimension; LVEF, left ventricular ejection fraction.

* Weighted means for medians across study level groups.

* Six hundred forty‐five patients analyzed with available data in the modified intention‐to‐treat population.

* LVEF <40% and LA >50 mm were exclusion criteria.

* One hundred eighty‐seven patients in sinus rhythm included in the analyses of AF recurrence and 204 patients included in baseline descriptives, among 214 randomized in the trial.

* One hundred ninety‐nine patients were analyzed among 205 originally randomized (6 patients refused cardioversion and were excluded from analyses).

* Four patients had electrical cardioversion cancelled and were excluded from analyses.

* Left atrial area.

* LVEF ≤55% in 8.3% (n=9) of patients, LA ≥2.3 cm/m^2^ in 4.9% (n=5) of patients.

Median Jadad's score was 4 points, with 3 reports having <3 points.^37–38,45^ Studies evaluating n‐3 PUFAs to prevent recurrent AF had higher scores than postoperative studies (median, 5 versus 4 points, respectively).

### Patients

Table 2 shows demographic, clinical, and echocardiographic characteristics of study participants. Most patients were male, and the mean age ranged between 55.5 and 69.5 years. There was high prevalence of hypertension, diabetes, and previous myocardial infarction or coronary artery disease. Concomitant therapy with β‐blockers ranged from 25.5% to 84.5%. Use of amiodarone was an exclusion criterion in 8 studies and mandatory in 2 and varied from 3.8% to 63% in the remaining studies (1 study, reported as proceeding abstract, did not report this information). Mean left atrial dimension (or area) was mildly dilated in most studies, and mean left ventricular ejection fraction was in the normal range.

### Outcomes

#### Maintenance of sinus rhythm after AF reversion studies

**Figure 2.**
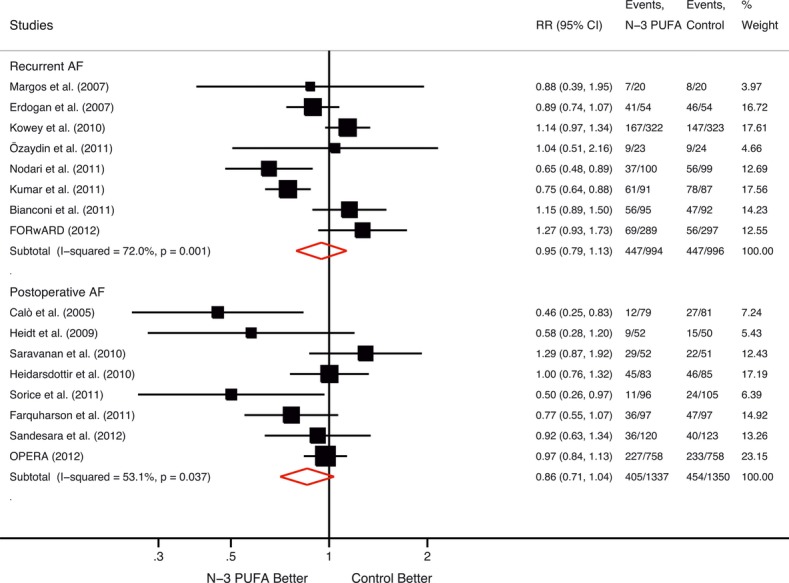
Effects of n‐3 PUFA on AF. PUFA indicates polyunsaturated fatty acid; AF, atrial fibrillation; RR, relative risk.

**Figure 3.**
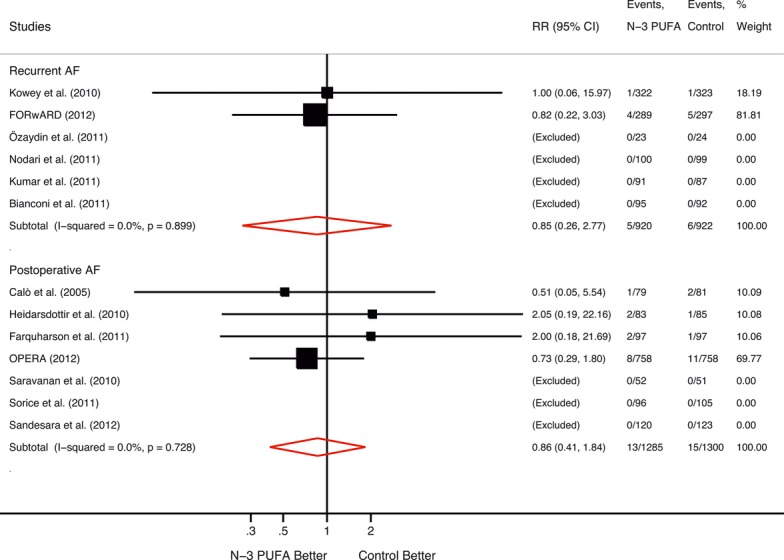
Effects of n‐3 PUFA on mortality. PUFA indicates polyunsaturated fatty acid; AF, atrial fibrillation; RR, relative risk.

Figure 2 shows the cumulative results of n‐3 PUFAs on AF. Treatment had no effect on AF recurrence (RR, 0.95; 95% CI, 0.79 to 1.13), with moderate inconsistency across trials (I^2^, 72.0%). Overall death rates were low among the 5 studies that reported this information, and there was no effect of n‐3 PUFAs on mortality (RR, 0.85; 95% CI, 0.26 to 2.77; Figure 3).

#### Postoperative studies

Among studies evaluating the effects of n‐3 PUFAs to prevent postoperative AF, treatment resulted in nonsignificant reduction of AF during hospitalization (RR, 0.86; 95% CI, 0.71 to 1.04), with moderate inconsistency (I^2^, 53.1%) (Figure 2). There were no effects on in‐hospital death across the 6 studies that reported this outcome (RR, 0.86; 95% CI, 0.41 to 1.84; Figure 3).

**Figure 4.**
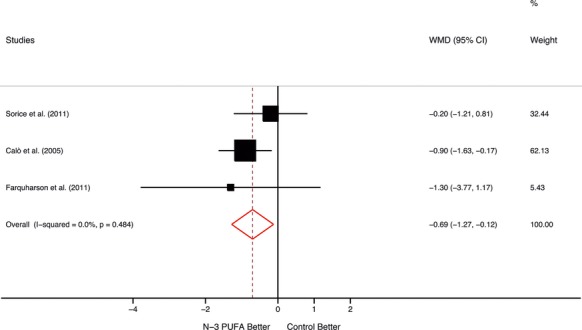
Effects of n‐3 PUFA on length of stay among postoperative AF trials. PUFA indicates polyunsaturated fatty acid; AF, atrial fibrillation; WMD, weighted mean difference; CI, confidence interval.

Three studies reported information on ICU length of stay. Treatment with n‐3 PUFAs resulted in significantly shorter stay compared with the control (weighted mean difference, −0.69; 95% CI, −1.27 to −0.12), with low inconsistency across trials (I^2^, 0.0%) (Figure 4).

### Publication Bias

**Figure 5.**
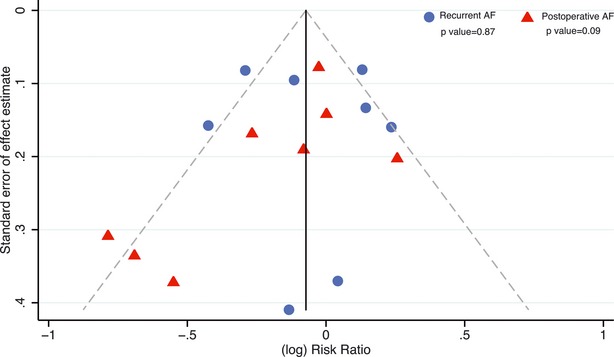
Publication bias assessment. AF indicates atrial fibrillation.

Figure 5 shows a funnel plot. Formal testing of publication bias showed no evidence of bias among studies evaluating n‐3 PUFAs to prevent recurrent AF (*P*=0.87). Visual inspection of the funnel plot and formal testing of its asymmetry showed evidence of publication bias among studies assessing effects of n‐3 PUFAs to prevent postoperative AF (*P*=0.09).

### Sensitivity Analyses

**Figure 6.**
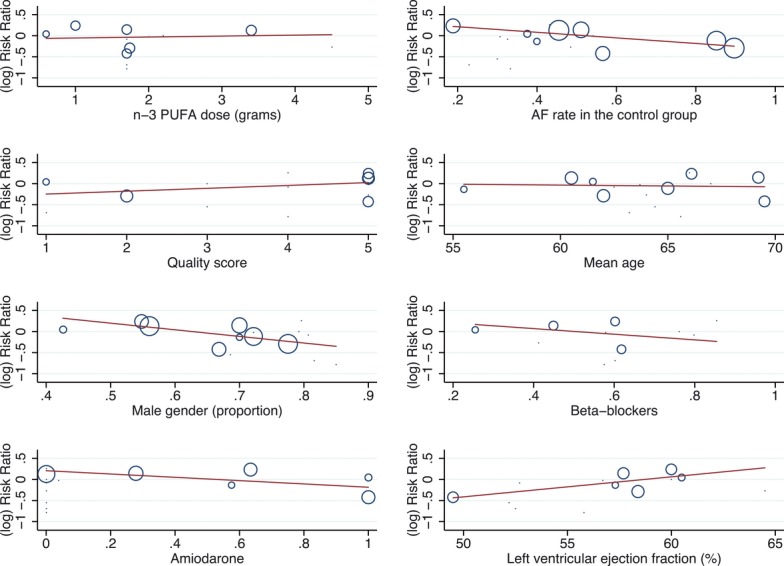
Meta‐regression of recurrent AF studies. AF indicates atrial fibrillation; PUFA, polyunsaturated fatty acid.

**Figure 7.**
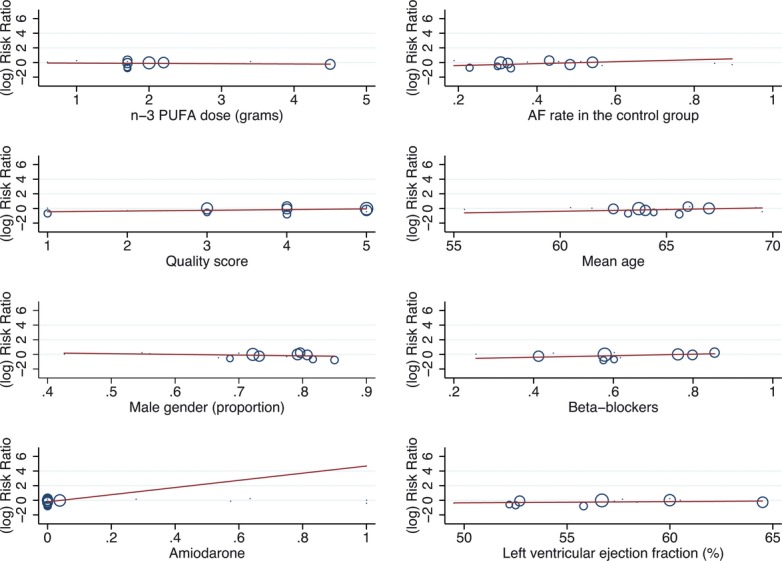
Meta‐regression of postoperative AF studies. AF indicates atrial fibrillation; PUFA, polyunsaturated fatty acid.

Repeated analyses excluding studies with a quality score lower than the median did not materially change the results. For AF recurrence studies, the risk ratio was 0.99 (95% CI, 0.83 to 1.20), and for postoperative AF, it was 0.89 (95% CI, 0.71 to 1.12). Other sensitivity analyses, including previous/concomitant use of β‐blockers, amiodarone, age, and sex did not provide different results (Table 3). Meta‐regression analyses suggested that the dose of n‐3 PUFAs did not influenced their effects (for recurrent AF, *P*=0.887; for postoperative AF, *P*=0.833). Additional meta‐regression analyses did not find associations between several study‐level covariates and the effect‐size estimates (Table 4 and Figures 6 and 7).

**Table 3.** Sensitivity Analyses

Characteristics	Recurrent AF			Postoperative AF		
	n/Events	Pooled RR (95% CI)	I^2^ (%)	n/Events	Pooled RR (95% CI)	I^2^ (%)
Quality score						
<4	225/157	0.76 (0.65 to 0.89)	0.0	471/150	0.72 (0.44 to 1.17)	59.1
≥4	1765/743	0.99 (0.83 to 1.20)	65.9	2206/709	0.89 (0.71 to 1.12)	58.6
Age, y						
<64.2	910/486	0.93 (0.68 to 1.28)	77.7	2154/654	0.86 (0.70 to 1.06)	39.7
≥64.2	1080/408	0.96 (0.74 to 1.24)	73.8	533/205	0.83 (0.54 to 1.26)	69.7
Male sex						
<72%	1704/668	1.03 (0.83 to 1.27)	58.5	102/24	0.58 (0.28 to 1.20)	*
≥72%	286/226	0.81 (0.68 to 0.96)	48.8	2585/835	0.88 (0.72 to 1.06)	55.0
β‐Blockers						
<60%	234/121	1.14 (0.89 to 1.46)	0.0	1870/582	0.77 (0.54 to 1.09)	70.6
≥60%	1756/773	0.91 (0.74 to 1.12)	77.4	817/277	0.90 (0.68 to 1.19)	49.4
Amiodarone						
<58%	872/432	1.13 (0.99 to 1.30)	0.0	2687/859	0.86 (0.71 to 1.04)	53.1
≥58%	1117/462	0.86 (0.70 to 1.06)	66.2	—	—	—

AF indicates atrial fibrillation; RR, relative risk; CI, confidence interval.

* Only 1 study in the stratum.

**Table 4.** Meta‐regression Analyses

Covariates	Recurrent AF			Postoperative AF		
	Coefficient (95% CI)*	*P* Value	Residual I^2^ (%)	Coefficient (95% CI)*	*P* Value	Residual I^2^ (%)
n‐3 PUFA dose	1.02 (0.67 to 1.57)	0.891	80.7	0.96 (0.68 to 1.36)	0.782	59.4
AF rate in control group	0.53 (0.31 to 1.04)	0.070	40.2	3.73 (0.23 to 60.60)	0.292	58.5
Quality score	1.07 (0.86 to 1.33)	0.433	69.7	1.10 (0.85 to 1.43)	0.385	56.4
Mean age	1.00 (0.94 to 1.06)	0.877	75.8	1.05 (0.86 to 1.28)	0.572	58.4
Male sex*	0.21 (0.04 to 1.25)	0.075	44.6	0.38 (0.01 to 260.19)	0.727	58.7
β‐Blockers*	0.51 (0.01 to 294.11)	0.695	77.5	2.85 (0.44 to 18.50)	0.209	51.4
Amiodarone*	0.68 (0.35 to 1.30)	0.173	39.4	134.10 (0.01 to 510.10)	0.566	56.4
LVEF	1.05 (0.96 to 1.15)	0.222	68.0	1.02 (0.95 to 1.09)	0.578	57.6

AF indicates atrial fibrillation; CI, confidence interval; LVEF, left ventricular ejection fraction.

* Coefficients express the change in the (log) risk ratios for every increase in 1 unit in the value of the covariates.

* Proportions in every study.

## Discussion

The present systematic review was sufficiently powered (93% of power at the conventional type I error level of 0.05 to detect a 20% reduction in recurrent AF and 88% to detect a 15% reduction; these numbers for postoperative AF were 96% and 80%, to detect 20% and 15% reductions, respectively) to close the uncertainty that existed regarding potential effects of n‐3 PUFAs for the prevention of AF.

The data obtained with >4500 patients and 1753 events regarding the efficacy of n‐3 PUFAs in preventing recurrent AF showed that the effect of pharmacological supplementation with these compounds resulted in no benefit. That these results were obtained evaluating studies that included patients who were receiving amiodarone or β‐blockers and others that excluded these treatments and across a wide range of doses of n‐3 PUFA strengthen the main finding.

In the setting of secondary prevention of AF and beyond the heterogeneity observed in clinical trial designs, the number of patients and events collected in this meta‐analysis allows confidence in the main conclusion of neutral effects of n‐3 PUFAs for this clinical indication. Moreover—and for reasons that remain unclear—the 2 most recent trials conducted, which contributed a large number of patients and events,^36,39^ showed an excess accumulation of AF among patients randomized to n‐3 PUFAs compared with those assigned to placebo. When separately considering the results of individual clinical trials, it seemed appropriate to propose a large, “definitive” randomized trial, but this meta‐analysis would call for caution. The economic and logistic effort to conduct such a trial would be substantial, and the cumulative results seem to be confirmatory of no benefit.

Similarly, results obtained for the prevention of postoperative AF have also been disproved according to this analysis. The incorporation of a large and well‐conducted clinical trial^47^ provided strength to this systematic review, a characteristic that was not present in previous meta‐analyses.^23–25^

Other systematic reviews had appraised the evidence on the effects of n‐3 PUFAs to prevent AF^23–25,48^ with no definitive results, but our analysis, which more than doubled the number of patients and events, precludes any beneficial effects of n‐3 PUFAs for the prevention of secondary and postoperative AF.

Trials had different designs, and this resulted in an important degree of heterogeneity. Of note, the doses of n‐3 PUFAs varied nearly 10‐fold across studies. The present analysis also explored whether the effects of n‐3 PUFAs on AF prevention would vary with the dose used in different trials. The result of the meta‐regression does not support the hypothesis, as no relationship was observed between dose and effect. However, it also should be noted that the studies were too few to draw any definitive conclusion regarding the dose effect.

Besides AF prevention, cumulative results also failed to demonstrate any benefit of n‐3 PUFA supplementation on other relevant end points, including mortality, although all trials were underpowered to detect differences on this end point.

It is important to note that these trials were restricted to a particularly high‐risk population (ie, those under secondary prevention and those undergoing cardiovascular surgery), and these results should not be extrapolated to a potential beneficial effect of these compounds in the context of primary prevention of AF.

In addition, the trials had an inherent short duration. It could be the case that n‐3 PUFA supplementation would require a more prolonged duration of exposure to see a potential benefit.

In conclusion, the present meta‐analysis provides confident evidence of the lack of usefulness of oral supplementation of n‐3 PUFAs for the secondary prevention of AF and for the incidence of new AF in patients undergoing cardiovascular surgery.
